# Skin-on-chip: Quo vadis?

**DOI:** 10.1063/5.0268706

**Published:** 2025-10-20

**Authors:** Mina Ghiță-Răileanu, Bianca-Maria Tihăuan, Irina-Oana Lixandru-Petre, Georgeta-Luminița Gheorghiu, Gratiela Gradisteanu Pircalabioru, Gabriela Cioca, Florina S. Iliescu, Ciprian Iliescu

**Affiliations:** 1 eBio-hub Centre of Excellence in Bioengineering, National University for Science and Technology, Politehnica Bucharest, Bucharest, Romania; 2 Academy of Romanian Scientists, Bucharest, Romania; 3 Research Institute of University of Bucharest, University of Bucharest, Bucharest, Romania; 4 Lucian Blaga University of Sibiu, Sibiu, Romania, Romania; 5 National Research and Development Institute for Microtechnology, IMT Bucharest, Bucharest, Romania; 6 National University for Science and Technology Politehnica Bucharest, Bucharest, Romania

## Abstract

There is a need for reconstructing the structural and functional complexity of human tissues such as skin to replace the animal models and provide accurate knowledge while solving ethical challenges in human medicine. Lately, microfluidics and tissue engineering have significantly advanced the development of 3D cell cultures and skin-on-chip (SoC), thus offering a cost-effective alternative to the generally used preclinical drug screening, toxicology applications, and cosmetic testing models. The current work presents a critical view on the SoC, from the fundamental concepts to the fabrication, applications, and commercialization. It comprehensively discusses the challenges faced by the 3D skin models and the perspectives of microphysiological skin platforms for preclinical pharmaceuticals and cosmeceuticals screening and disease research. It also highlights the technical and ethical requirements for successful SoC as physiological and pathological models applicable to personalized medicine. The SoC clinical and commercial translation depends on developing convergent biomanufacturing strategies and infrastructure focused on applications such as personalized skin disease models, skin grafts, and drug or cosmetics screening platforms.

## I. INTRODUCTION

It is acknowledged that drug or cosmetics research and marketing are prolonged and expensive, and launching a new formulation is estimated at 2.6 billion euros.^1^ Moreover, there is a lack of correlation between the pharmaceutical industry's input and output, probably due to significant imperfections in testing methods, especially after preclinical development and the start of clinical trials. Conventional preclinical drug testing depends on *in vitro* cell cultures and animal models. Although animal models support finding essential pharmaceutical solutions regarding drugs systemic effects, they cannot replicate human physiology,^2^ suffer from low output and repeatability, and present interspecies variability.^3^ Furthermore, they represent significant ethical challenges. Generally, *in vitro* cell culture relies on 2D arrangements of cells (cell monolayers). While these models help assess compound cytotoxicity,^4^ they are imperfect predictors of complex interactions observed *in vivo.*^5^ A step forward is obtaining 3D cell culture models (spheroids or organoids—single or multiple types of cells assembled in the same 3D structure) that better mimic *in vivo* conditions through increased cell-cell and cell-matrix interactions.^2^ Nevertheless, the development of complex biomimetic organ *in vitro* models, such as organ on a chip (OoC) where 3D cell culture models are under perfusion, can be a step forward in the prediction of drug toxicity and efficiency in preclinical studies with effect on cost and time required for bringing a new drug on the market.^6^

**FIG. 1.**
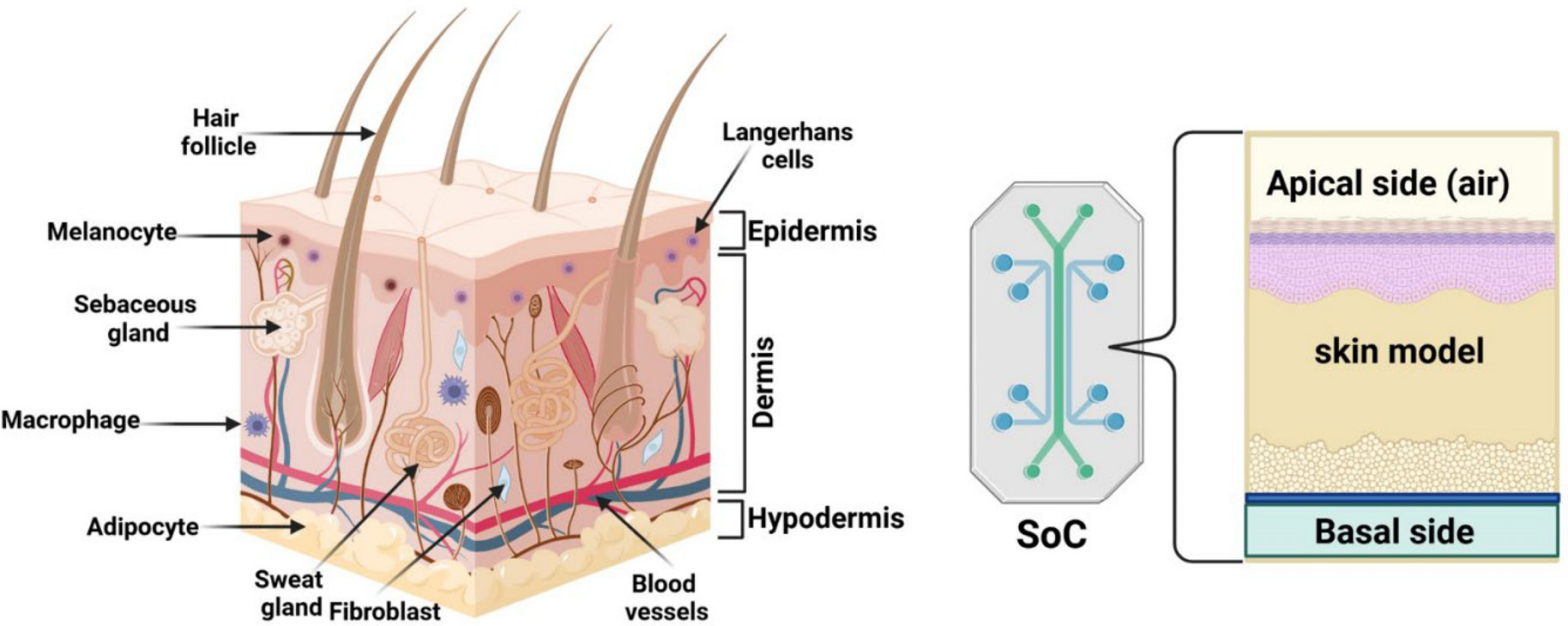
From skin structure to Skin-on-a-Chip model.

As the largest human organ, the skin research requires new approaches to treat skin maladies^7^ (Fig. 1). Psoriasis, atopic dermatitis, and eczema, considered chronic skin diseases, have significant morbidity cases reported^8,9^ and can affect the patients′ quality of life.^10^ In contrast, malignant skin diseases such as malignant melanoma are statistically fatal.^11^ Nevertheless, the cosmetic industry addresses various populations and requires specific human skin models for preliminary testing. Most human skin models are cultured under static conditions, enabling good modeling of physiological conditions and limiting the evaluation of pharmaceutical or cosmetic compounds in terms of delivery, efficiency, or toxicity.

The current work presents an overview of the state-of-the-art microphysiological systems targeting human skin models (skin-on-chip) for *in vitro* testing of pharmaceutical or cosmetic compounds. The promising results (the goods), the challenges (the beads), and the perspectives of this research field are also presented to depict an era of technological advancements toward personalized and integrated medicine. The recent developments focused on applications are presented and explain the SoCs′ (skin-on-chip) potential as physiological and pathological models to replace the animal models in pharmaceutical and medical research.

## II. UNDERSTANDING CHALLENGES AND THE COMPLEXITY OF HUMAN SKIN – THE GOOD, BAD AND UGLY FACETS FOR SKIN MODELS

**FIG. 2.**
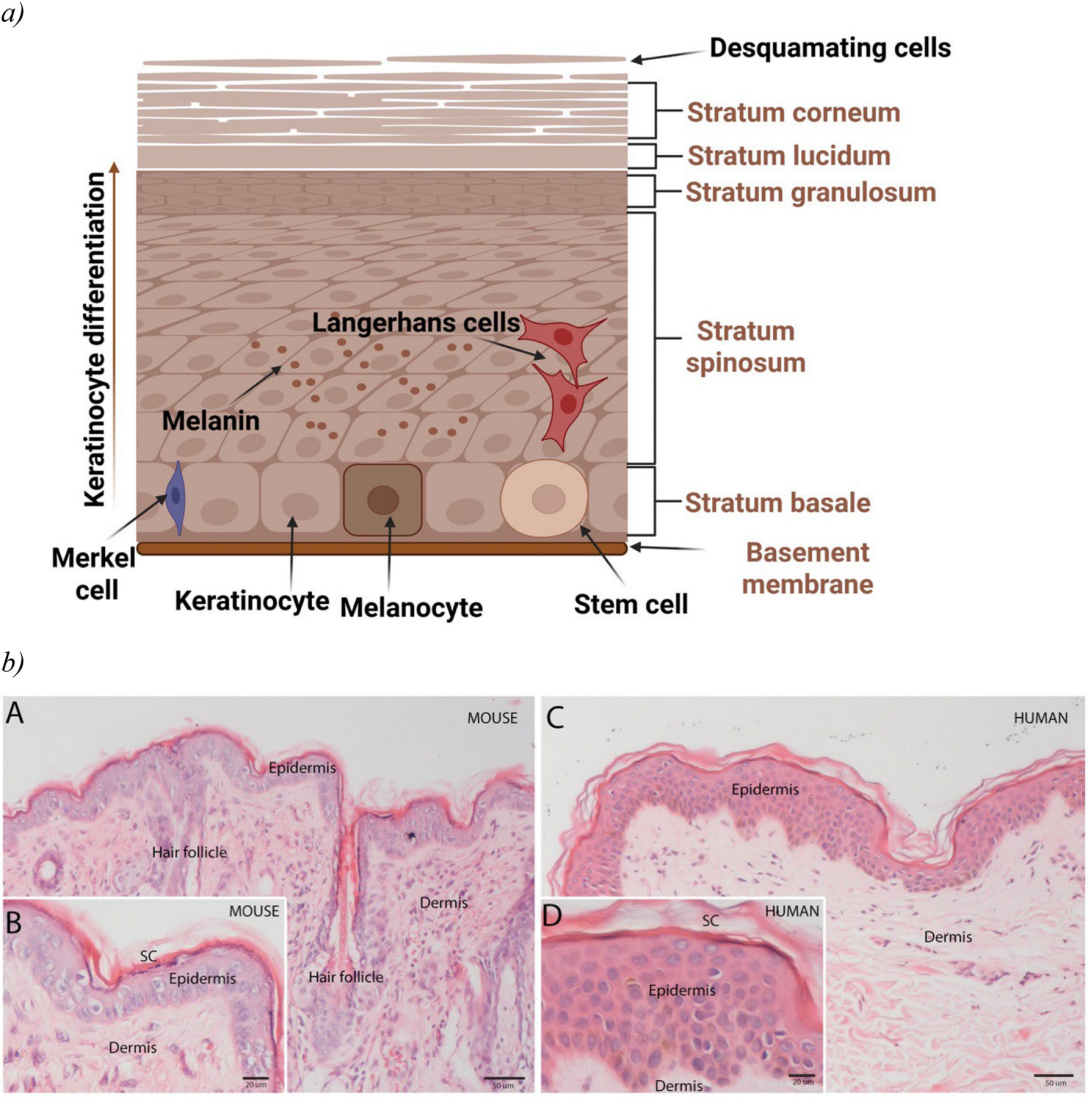
(a) A schematic representation of human skin-original figure created using Biorender; (b) Human vs mouse skin (Epidermis and Dermis) within the integumentary system. Epidermis is thicker in human skin than in mouse; more hair follicles in mouse skin than in human [(A) and (C) 10X]; [(B) and (D) 20X]; SC-Stratum Corneum (Reprinted with permission from Salgado *et al.*, Differentiation **98**, 14–24 (2017). Copyright 2017 Elsevier^20^).

As the largest human organ, the skin plays a vital role in human body′s homeostasis, from protecting to regulating the biological microenvironment.^12^ The skin within the integumentary system acts as a complex barrier, reducing to maximum any damaging physical, chemical, or biological invasions while acting as a selective physiological membrane involved in active metabolic processes. Skin, an essential interface with the external environment, also acts as one complex biosensor involved in thermoregulation and sensorial functions.^13^ Moreover, annexes such as hair follicles, glands (e.g., eccrine sweat, sebaceous, apocrine), along with heterogeneous cells (fibroblasts, keratinocytes, melanocytes, Langerhans, and Merkel cells), and extracellular constituents build this complex barrier. Notably, the human skin′s topographical intra- and inter-variability, such as thickness (the eyelids 0.5 mm—the heels 4.0 mm), cellular composition (keratin, melanin), density of extracellular structures (collagen and elastin fibers), and biochemical differentiation (division rate and metabolic activity) are essential when defining the normal skin and evaluate skin-related diseases.^14^ Such variability has a significant impact on the biological response and external input outcomes: it could act either as a “friend” when providing specific and distinct mechanical features or a “foe” when delaying the absorption of medicated formulations or design of skin models and implicitly the analysis of causes, risk factors of skin diseases and effective therapeutic schemes. For instance, the stratum corneum, the skin's superficial layer [Fig. 2(a)], comprises 20–30 layers of flattened dead keratinocytes (corneocytes), which contain mostly keratin and are continuously discarded and replaced by cells from the deeper layers. The protein-rich content and the extracellular lipid structure make a “brick-and-mortar” structure, protecting deeper layers from injury and microbial intrusion. Meanwhile, drug delivery through stratum corneum is a real challenge as it is impermeable to hydrophilic molecules and allows passage only to very small, highly lipophilic molecules.^15^

Furthermore, there are multiple bonds between the various cells in adjacent layers. Highly crosslinked proteins compactly bound to lipids form a cornified lipid envelope within the wall of corneocytes. Then, the tonofilaments attached to desmosomes and hemidesmosomes maintain the integrity and stability of the epidermis and dermis: they connect the cells in the *stratum basale* one to another and with the cells with the adjacent *stratum spinosum* and the keratinocytes to the basement membrane. Intra- and interlayer cellular interaction, adhesion, proliferation rate, differentiation, and apoptosis determine epidermal homeostasis and the normal and abnormal skin. Thus, skin diseases can be studied through physiological variations. However, the complexity makes the understanding of pathophysiology and the modeling difficult [Figs. 2(b-A) and 2(b-D)]. For instance, the reconstructed human epidermis (RHE) includes human isolated-from-donors keratinocytes. While essential epidermal differentiation markers such as K1K10 and K2e are common, RHE lacks genes related to intercellular interactions, such as the keratinocytes-fibroblasts crosstalk.^16^ Therefore, reconstructing the human epidermis from the isolation and culturing to raising to the air–liquid interface for keratinocyte differentiation requires knowledge of the histology of human skin transcriptomes for quality assurance, quality control, and standardization. Moreover, the commercially available models must be accompanied by detailed scripts about the types of cells, construct, histology, immunohistochemistry, lipid analysis, permeability, toxicity testing, and cell handling.^17^

The dermis consists of fibrous and elastic connective tissues, with a non-fibrous component such as fine filaments, glycoproteins, proteoglycans, and glycosaminoglycans. The woven network of fibers in the dermis provides the skin′s great tensile strength (opposes pulling or stretching forces) and the capacity to stretch and recoil easily.^18^ Type I collagen fibers offer the greatest mechanical robustness. For instance, the papillary dermis small-diameter collagen fibrils provide the primary defense against mechanical stress. In contrast, the reticular dermis, with interwoven bundles of large-diameter collagen fibrils, makes the skin elastic and mechanically resilient.^19^ The dermis capillary network is essential to epidermis homeostasis, while the dendrites contribute to an active response to environmental changes through the sensory system. The free-nerve endings trigger nervous action potentials within the sensory system for feelings such as heat, cold, pain, tingling, and irritation.

Skin is an integrative system with physical, chemical (e.g., metabolic, excretion), and sensorial-based protection, thermoregulation, storage capabilities (e.g., blood), and support for a balanced state essential for a healthy life. Disruption of this system can lead to diseases. For instance, when large and deep enough skin injuries destroy the regenerative layer *stratum basale*, new skin cannot be formed, and the repair mechanisms lead to scars as improper patching tissues with less protective abilities. When skin wounds of magnitude require more than skin grafts to heal, autologous skin transplantation employs the individual's epidermis cultured keratinocytes to produce transplantable thin sheets of skin (e.g., Apligraf, Transit) for the patient. This process is challenging, involving accurate histology and physiology knowledge, technical skills for specific procedures, and up-to-date technology [Fig. 2(a)] to achieve the correct and beneficial outcomes. This also implies knowledge about animal models [Figs. 2(b-A) and 2(b-B)] and how to differentiate from the human skin [Figs. 2(b-C) and 2(b-D)].

Unique challenges stem also from the complex and in-relative-motion interacting surfaces within the integumentary system (skin tribology) applied to studying skin diseases and transcutaneous drug delivery. For instance, applying skin creams and the associated skin-product interactions can alter the skin's tribological properties, leading to lower product efficacy. These challenges underscore the need for continuous engagement and innovation in the dynamic field of skin biology. It is not just a necessity, but also an exciting journey of discovery.

Since several aspects are valuable during evaluating the skin's biomechanics^21^ such as skin layer, topographic anatomy, age, skin hydration, mechanical loading type/period or direction, and strain rate,^22^ there are many characteristics to be investigated, such as surface roughness, friction, adhesion, elastic modulus, and surface charge.^23^ Such diversity is the challenge, and the case of *stratum corneum* is a good example. This layer controls the percutaneous passage essential for chemical absorption or excretion through its natural key lipid elements. The ceramides (CER), cholesterol, cholesterol sulfate, and free fatty acids (FFA) from lamellar membranes are templated by the patterned corneocyte wall^24^ and extend horizontally parallel to the corneocyte.^25^ Moreover, the metabolically active extracellular matrix with enzymes, structural proteins, and antimicrobial peptides has its role as a dynamic barrier.^26^ It can be argued that cosmetics or medicated ointments interact with the epidermal lipids and mimic how sebaceous glands-secreted triglyceride incorporate the epidermal lipids and contribute to the skin lipids fluidity and barrier functions.^27^ Therefore, its reconstruction must follow the skin barrier's natural elements and variations, such as the changed amount of native human CER 1 and 4.

Apart from the structural elements, the functional metabolic aspects added to the challenges. The absorbed substances may enter the skin cells and be subjected to local cellular metabolism like the digestive system first pass effect. Even though the transformation is significantly less intense, skin cells′ enzymatic package could interfere with the physicochemical properties of the absorbed substances and influence pharmaco-dynamics and pharmaco-kinetics, from efficacy and potency to elimination and toxicity. Metabolic phases lead to functionalization and conjugation of the active ingredients or adjuvants in the formulations and significantly alter the dermal pharmacokinetics profile.

Furthermore, when frozen tissue specimens are considered for *ex vivo* testing, the local metabolism cannot be fully recovered, and more challenges add to the modeling process. Therefore, local metabolism influences the permeation potential and is essential when modeling skin.^28^ This is also the case when data from *ex vivo* or life animal-based tests are compared with the tissue models.

In the case of the skin within the integumentary system, understanding the specific role of the subcutaneous tissue is an advantage in reconstructing the complete models for pathophysiology.^29^ Focused work in this area is essential to unlocking a deeper understanding of skin health and disease. Cells other than adipocytes (ASCs, endothelial cells, smooth muscle cells, and macrophages) are possibly beneficial additions to the HSE.^30^ Therefore, it is essential to have commercially available living skin equivalents to analyze the relationship between the epidermis and the dermis. Ideally, models like the Phenion Full-Thickness (FT) with improved predictive power compared to traditional *in-vitro* methods derived from using human skin cells in a 3D environment, which well reflects human native skin.^31,32^

Moreover, it is acknowledged that distinctive animal models have been used for research. For instance, mouse models are often mandatory for *invivo* translational research of skin physiology, biochemistry, and immunology in various pharmacological and toxicological studies because of their ease of handling in the laboratory. However, comparative studies showed significant distinctions between mouse and human skin. Noticeable was the fact that pharmaceutical absorption studies performed on non-human skin (murine and porcine) do not always reliably predict the outcome on human skin due to inter-species differences. Murine skin is structurally and functionally different: it presents more skin appendages (more hair follicles), fewer epidermal and keratinocyte layers, and is loosely attached to the underlying muscular layers,^33^ with a reduced barrier function and better absorption.^34^

Furthermore, the mouse skin immune system includes different dominant T cells and arrays of expressed chemokines. Such differences make mouse models poor predictors of the human skin response to topical active substances, leading to either unsuccessful clinical trials or disqualifying others potentially effective in humans. Also, despite closer to human skin morphology, physiology, and repair mechanisms, the porcine skin presents specific differences that hinder the translatability of animal-based studies.^35^ Addressing the challenges via modeling human skin *in vitro* would improve this translatability and reduce animal experiments-based preclinical evaluations. Moreover, there is an opportunity to explore alternative solutions to animal models and propose biomimetic skin modeling to reproduce various skin key components and address the need for *in vivo* skin-based experiments in developing and testing new compounds for cosmetic and pharmaceutical applications.

Compared to its mammalian counterparts, human skin's unique characteristics, such as pigmentation, dermal evolution, adipose tissue presence, and skin appendage, decide when skin behavior and possible associations between species become inaccurate. Notably, the cellular interactions at various layers within the skin control the skin's response to certain materials and stimuli. Thus, it is challenging to predict the passage or absorption of chemicals within several materials into the human skin by studying these processes on other mammals' skin with different structures and architecture. This is the case in cosmetics development, which still employs animal model testing methods. To address this, merging 3D bioprinting and SoC might become the modality to create fully functional skin models for cosmetic and drug testing. However, difficulties are foreseen in bioengineering physiologically relevant skin models as the skin's functional and architectural complexity stems from its specialized cells and physiological subsystems.

Having skin models as biomimetically designed alternatives requires attentive consideration of various chemical and physical factors that allow the fabricated models to imitate the structural and mechanical features matching the specific clinical and pharmacological parameters and to toxicological test for skin irritation, permeation, corrosion and hydration, genotoxicity, and absorption^36^ in close-to-life conditions. From an ethical standpoint, however, replacing the animal models answers a growing societal demand concerning animal-based research. Ethical guidelines based on the 3Rs principle and applied within the EU limit, where possible, animal experimentation since 2009.

Within the EU, The European Commission approved regulations on cosmetics and established animal testing and marketing ban on cosmetic formulations or ingredients.^37^ The combinatory effect of the imposed restrictions, increasing prevalence of skin diseases, and decreasing R&D stressed the need for physiologically relevant skin models that could replace conventional, inefficient models. To answer the demand and support patient-oriented research while respecting the globally accepted “3Rs” (Replacement, Reduction and Refinement) principle of humane animal research,^38^ promising skin-on-a-chip (SOC) and organ-on-a-chip technology emerged for 3D skin models with the help of 3D bioprinting technology.^39^ Improving the 3D culturing techniques increased the available models' relevance and demonstrated the synergy between different cell types.^40^ 3D complexly assembled models could simulate more physiologically relevant conditions and cell-matrix interactions, reconstitute human skin, and open new avenues for investigating cancer immunology,^41^ skin pathologies and developing new therapeutic agents.^42^ Moreover, advances in tissue engineering permitted a close recapitulation of the native human skin functions and have been used for fundamental, pharmacological, and cosmetic research. The SoCs' precise applications relate to specific experiment design and interpretation and require modeling different skin layers based on the complex synergistic effects between epidermal keratinocytes and dermal fibroblasts^43–45^ and the hypodermis adipocytes' role in the intercellular communication that stimulates fibroblast migration and wound healing.^46^

## III. DEVELOPMENT METHODS FOR 3D SKIN MODELS

### A. Cell sources for skin-on-chip

The cellular source highly conditions SoC biomimetic platforms, as attaining the desired architecture and physiology is vital. Over the past three decades, a deeper understanding of skin and adjacent tissue cytology pushed forward the progress of the SoC domain toward accomplished skin equivalents with various dynamic and vascularized models.^47,48^ However, miniaturized *ex vivo* platforms encountered several technological challenges stemming from inadequate surface relevance to function complexity ratios and related intrinsic variability. To date, among the several *in-house* and commercially available skin mimicking models is the reconstructed human epidermis (RHE),^49^ as a reliable test method for *in vitro* skin irritation (TG 439) implemented in 2010 by OECD guidelines through the European Center for the Validation of Alternative Methods (ECVAM) validation study.^50^ However, engineering 3D skin models from human primary cells is challenging mainly due to the misrepresentation or lack of immunological landscape.^51–54^ The limitations are prevalent among skin models, from reconstructed epidermis^51^ or models containing endothelial cells (EC)^55^ to full-thickness models^53^ and three-layer replicants.^54^ The cellular source can be autogenic (helps in minimizing immune rejection and enhancing the physiological relevance of the model), allogenic (contributes to a broader range of genetic diversity) or xenogeneic (considered when scarcity in a human cell is encountered or for interspecies interactions studies), each presenting specific advantages and challenges.^56^

The most reported cell types used in SoC applications are primary cells, standardized cell lines or induced pluripotent stem cells (iPSCs). Using primary human cells is considered conventional for SoC platforms at this point.^57^ The cells isolated from the healthy donors during standard surgical procedures can capture the *exvivo* phenotype, while the primary cells from donors with under-investigation diseases can be effectively employed for pathology models. For instance, some of the most attractive cells protocols for full-thickness skin models include an epidermis from primary keratinocytes and a dermal compartment comprising primary fibroblasts, with added melanocytes and Langerhans cells and lymphocytes.^58^ The essential benefits of using cell lines stemmed from the recognized reproducible, and reliable cell growth related protocols. Proof-of-concept models reported using primary human cells such as fibroblasts and keratinocytes, specifically primary dermal fibroblasts (NHDF), epidermal keratinocytes (NHEK), umbilical vein endothelial cells (HUVECs), normal neonatal epidermal keratinocytes (NHEK), HaCaT, fibroblasts (Hs27), dermal microvascular endothelial cells (HDMEC), follicle dermal papilla cells (HFDPC), and normal epidermal melanocytes (NHEMs).^57–59^ Table I presents the connection between the histology and possible SoC-related outcomes. The actual data show that improving the existing *in vitro* skin models implicates integrating adipocytes, dermal papilla cells to produce hair follicles, endothelial cells to promote vascularization, immune or Langerhans cells to replicate the immune response, chemokines to encourage cellular differentiation, and dorsal root ganglion neurons to reconstruct the peripheral skin nerve system. These additions help the models mimic the skin's representation more accurately in assessment of relevant responses to irritation or toxicity studies.^12,60^ While primary cells are invaluable for physiological and pathophysiological relevant SoC models, their use is limited by several factors. For instance, the proliferative capacity (senescence) and variability (donor differences and availability) limit lifespan and expansion potential and restrict cell use for long-term studies or large-scale applications. Moreover, technical challenges (especially isolation and purification) and possible immune responses generate further challenges in co-culture protocols. Notably, the ethical and regulatory constraints derive from all other challenges and, therefore, impose attentive experimental design, protocol standardization, and the development of alternative cell sources, such as iPSCs or immortalized cell lines, to complement primary cell-based models.

**TABLE I.** Microanatomy overview of normal skin for functional considerations of SoC.

Integumentary layers	Representative cells^61^	Associated ECM components^62^	Major structures^63^	Growth factors^64^
Epidermis	Keratinocytes, melanocytes, Langerhans cells, Merkel cells	Keratin, type IV/VII collagen (basement membrane)	Stratified squamous keratinized epithelium	epidermal growth factor (EGF), transforming growth factor beta (TGF-β), fibroblast growth factor (FGF), vascular endothelial growth factor (VEGF), and insulin-like growth factor 1 (IGF-1)
Dermis	Fibroblasts, endothelial cells, Langerhans cells, mechanoreceptor cells, Schwann cells, smooth muscle cells, hair follicle cells	Type I collagen, elastin, proteoglycans, type IV/VII collagen (basement membrane)	Blood vessels, nerves, mechanoreceptors, hair follicles, sebaceous glands, sweat glands	
Hypodermis	Adipocytes, fibroblasts, endothelial cells, smooth muscle cells, hair follicle cells	Type I collagen, elastin	Blood vessels, nerves, hair follicles	

### B. Skin-on-chip

One of the earliest functional SoC systems aimed at mimicking the skin layers with perfusion and helped replicate the barrier function. This work started with Eugene Bell's pioneering work in developing artificial skin.^65^ Currently, chip models allow researchers to study the permeability of different substances across the skin, offering a more realistic alternative to traditional *in vitro* models.

Growing biological barriers employing methods recognized as standards include permeable supports such as transwells to create two chambers and separate the tissue's apical and basal surfaces. For two reasons, such tissue separation is decisive during 3D skin modeling of a representative *in vivo*-like barrier. First, the compartmentalization seals the apical and the basal surfaces and distinguishes the air-liquid interface. Second, it paves the way for efficient application of the advances in biomaterials and microfabrication to the organ-on-a-chip (OoC) platforms and design new barrier models. For instance, inserting a semipermeable membrane to separate the elastomeric microfluidic channels and create domains within an OoC is considered a common and successful practice in constructing pulmonary, blood-brain barrier, intestinal, and hepatic biological barrier models.^66^

**FIG. 3.**
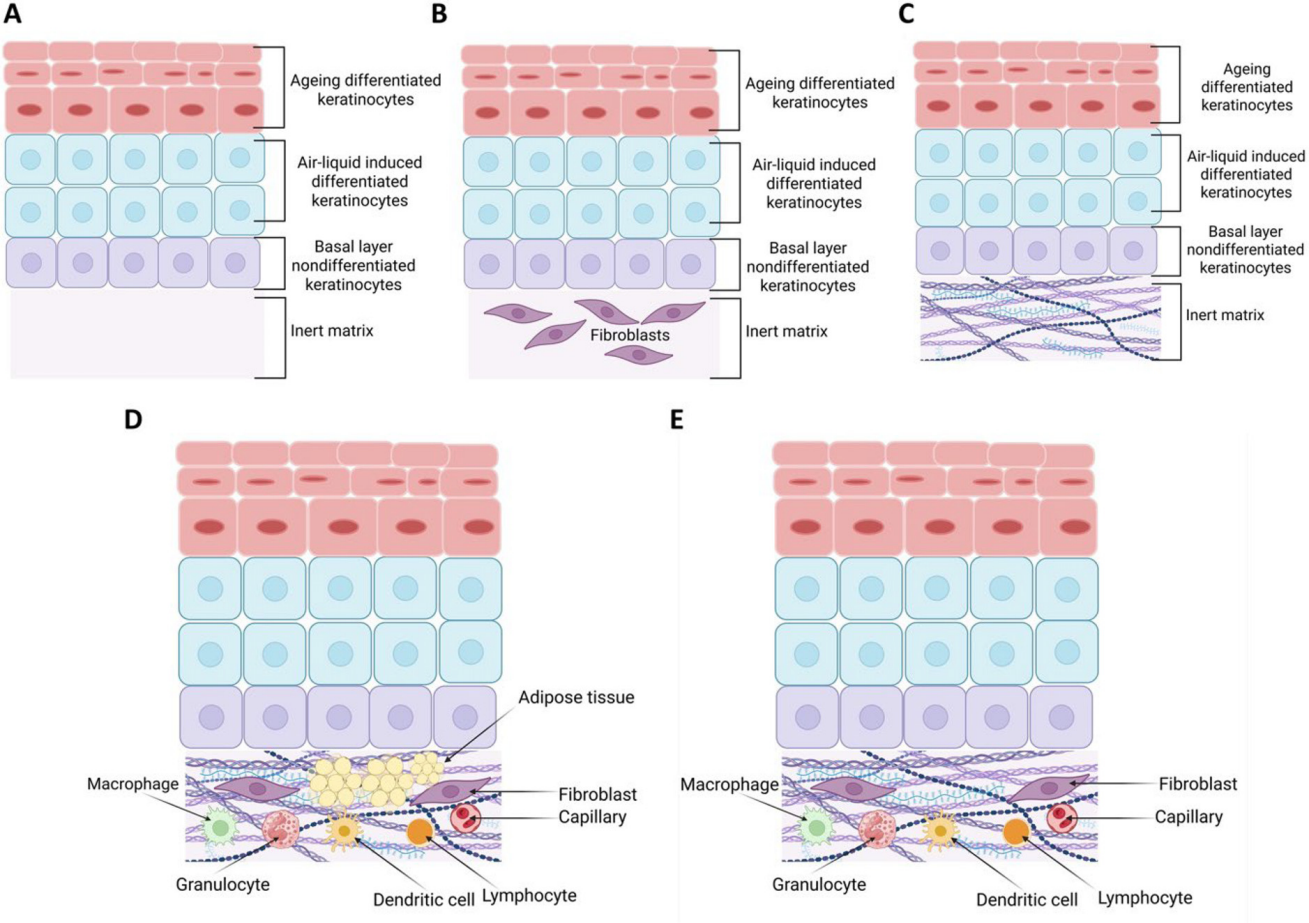
The progress of human skin equivalents. (A) a reconstructed human epidermis (RHE) model comprising keratinocytes seeded and cultured on an inert matrix then air-liquid-interfaced differentiated and stratified to resemble a physiological epidermis^67,68^ (B) a reconstructed human skin (RHS) as models comprising a dermal equivalent with fibroblasts grown in a synthetic matrix; (C) a RHS model comprising a dermal equivalent with fibroblasts on self-generated and -assembled collagen extracellular matrix (ECM)^69,70^ (D) a RHS with keratinocytes seeded on the dermal equivalent and ALI-induced differentiated.^71^ (E) the desired metabolically active integumentary model as close to native anatomy - skin (epidermis, dermis) and hypodermis (subcutis) equivalents with functionalized circulatory, immune and nervous components. Original figure created using Biorender.

**FIG. 4.**
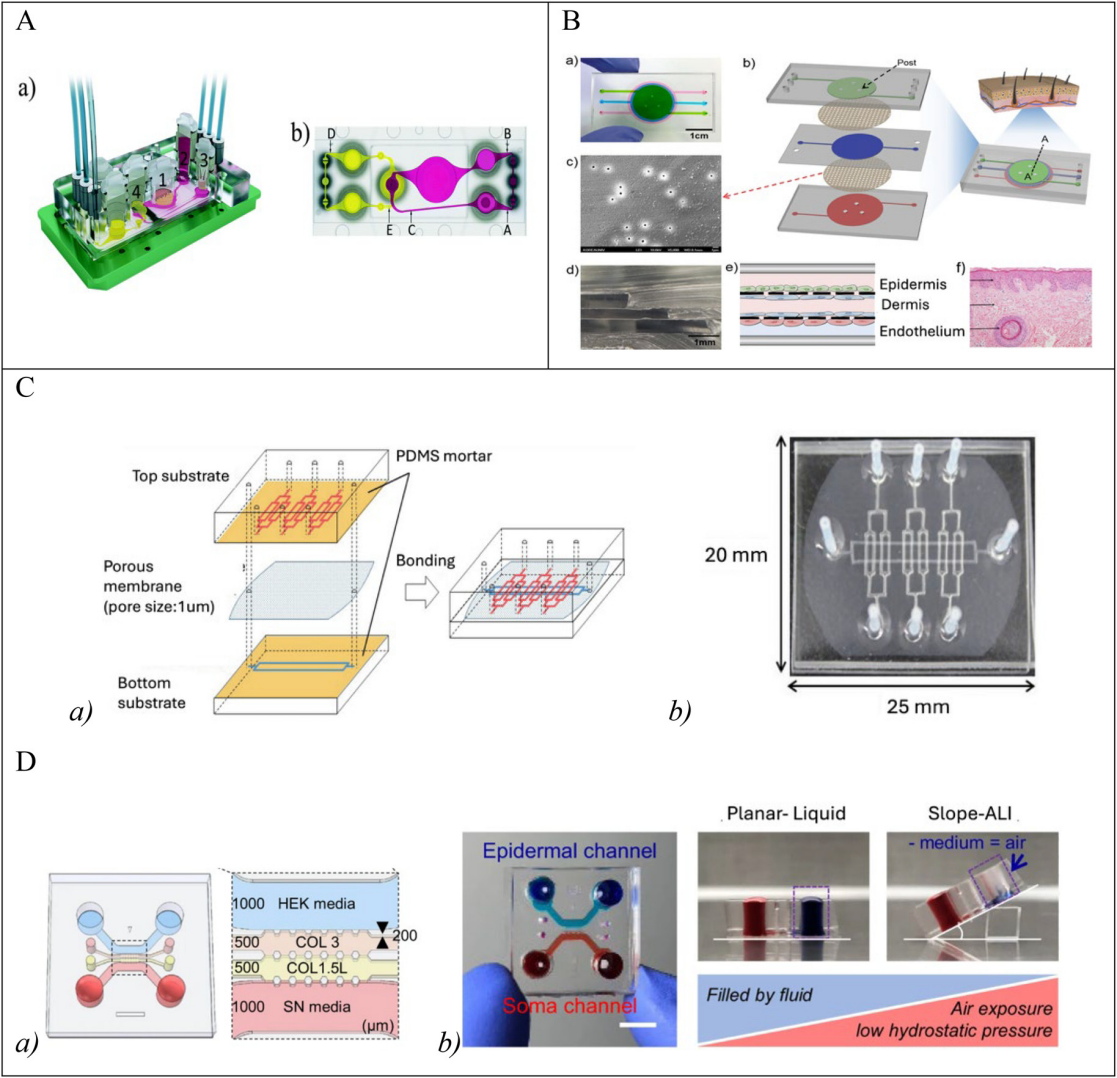
SoC types. (A) The microfluidic device: (a) with four-organ-on-a-chip (1) intestine, (2) liver, (3) skin, and (4) kidney; (b) the evaluation of fluid dynamics within the surrogate blood -A,B- and excretory -C,D,E- circuits; (c) the average volumetric flow rate. Reproduced with permission from Maschmeyer *et al.*, Lab Chip **15**(12), 2688–2699 (2015). Copyright 2015 Royal Society of Chemistry.^74^ (B) Image of a skin-on-a-chip device: (a) filled with fluid, (b) with three PDMS layers and two PET porous membranes, (c) the SEM image, (d) the cross-sectional image, (e) its schematic, including three separate channels with four vertically stacked cell layers, (f) the histological skin section stained with hematoxylin and eosin. Reproduced with permission from Wufuer *et al.*, Sci. Rep. **6**(1), 37471 (2016). Copyright 2016 Springer.^75^ (C) Schematic illustration of (a) fabrication of the microfluidic device and (b) its photographic image. Reproduced with permission from Sasaki *et al.*, Sens. Mater. **31**(1), 107–115 (2019). Copyright 2019 MYU K.K.^76^ (D) (a) *In vitro* innervated epidermal chip. (b) Top view of the microfluidic chip (left) and experimental concept of slope-based air-liquid interface (ALI) method for epidermal development (right, longitudinal vertical section view). Each cell channel was marked with a different color dye. (HEK-human keratinocyte, SN-sensory neuron, COL 3-collagen I at 3 mg/ml concentration, COL 1.5L- collagen I at 1.5 mg/mL with 10% laminin, Scale unit; *μ*m) Reproduced with permission from Ahn *et al.*, Nat. Commun. **14**, 1488 (2023). Copyright 2023 Springer Nature.^77^

However, it also presents essential limitations for developing complex 3D tissues, such as the full-thickness-skin model (FTSm). The dermal compartment must be generated for its development, which usually involves the polymerization of a hydrogel and cell mixture on top of a membrane.^72^ Creating the epidermis above (superficial) the dermis imposes two conditions. First, the apical chamber must support the precise construction of the stratified epithelium, air circulation or drugs for subsequent experimental phases. Second, the lower chamber must support the correct medium's perfusion. Notably, current approaches for integrating cell culture membranes rely on the irreversible bonding between the apical and basolateral culture microenvironments,^73^ influencing the ways the newly formed tissue is retrieved for structural and physiological analysis. FTSm generation presents limitations, such as challenging cell seeding, introducing a dermal matrix on the membrane, culture continuation, or accessing the tissue for end point analysis. Figure 3 represents how the *in vitro* modeling of human skin equivalents advanced from reconstructed human epidermis (RHE) toward a fully functional model. Figure 4 presents some of the SoC designs based of the fundamental model types described in the literature and indicated in Table II.

**TABLE II.** A summary of the SoC reported devices. Abbreviations: ECM, extracellular matrix; FTSm, full thickness skin model; HKs, Human keratinocytes; HFs, Human fibroblasts; HUVECs, human umbilical vein endothelial cells; iPSCs, induced pluripotent stem cells; PC, polycarbonate; PCL, polycaprolactone; PDMS, polydimethylsiloxane; PET, polyethylene terephthalate; PMMA, poly(methyl methacrylate).

Model type	Tissue (cell types)	Dermal matrix	Fabrication/materials	Main characteristics	References
Transferred skin-on-chip	Commercial FTSm (EpidermFT^®^)	Collagen	Lithography; PDMS + glass + PC	*Ex vivo* skin and hair follicle on a chip; prolonged lifespan	30
Transferred skin-on-chip	Human skin biopsy	Human skin biopsy	Lithography; PDMS	Neutrophil responses to *Staphylococcus aureus;* Skin infection studies	80
Multi organ-on-a-chip	Commercial TissUse'HUMMIMIC iPSCs and chips	Human skin biopsy	PDMS	Co-culture of skin and liver; Test exposure to troglitazone	81
Multi organ-on-a-chip	Commercial RHEm (EpiDerm^®^)	None	Commercial platform (TissUse'HUMMIMIC)	Co-culture of skin and liver; Pharmacokinetics (hyperforin, permethrin)	82
2D skin-on-chip	Cell monolayers (Immortalized HaCat HKs + Immortalized HS27 HFns + HUVECs)	Fibronectin coating	Lithography; PDMS + PET membrane	TNF-α induced skin inflammation; Simulation of skin edema	75
2D skin-on-chip	Cell monolayers (Immortalized HaCat HKs)	None	Laser cutter; PMMA + PDMS + PET membrane	Photolithography free; Irritation testing (potassium dichromate)	76
3D vascularized skin-on-chip	FTSm (Primary HKns + primary HFns) + iPSC-derived endothelial cells	Collagen	3D printing; Transwell inserts + PET membranes	Integration of iPSC; Promotion of neovascularization during wound healing in rat model	83
3D vascularized skin-on-chip	FTSm (Primary HKns + primary HFns) + HUVECs + Hypodermis model (primary HPAs)	Fibrinogen + dECM porcine skin	3D printing; PCL	3D cell printing; Integration of hypodermis	84
3D skin-on-a-chip	FTSm (Immortalized HaCat HKs + Primary HFs)	Fibrin	Edge plotter; PMMA + PDMS + Vinyl + PC membranes	Parallel flow method for bilayer tissue formation	85

If commerciality and large-scale production are not required, other alternative approaches can be considered for developing SoC devices. One alternative is establishing gel-liquid interfaces to physically support cells and an intercellular direct contact interaction. The interface is typically microfabricated using phase guides acting as capillary pressure barriers or using equally spaced post structures. This technique was used to construct a neurovascular unit that included human endothelial cells simulating the cerebral blood vessels and primary rat neurons and astrocytes.^78^ It is also possible to engineer embedded tubules in an ECM for SoC development. Homan *et al.* used bioprinting to generate convoluted tubules by printing a sacrificial ink embedded within the ECM.^79^ This structure modeled 3D renal tubules and recreated cyclosporine-dependent nephrotoxicity. Although these approaches have several limitations, including high production costs, the need for specialized personnel, and reproducibility concerns, they can be valuable tools for studying specific aspects of skin diseases and tackling the toxicology profile of drugs.

Moreover, to ensure global availability, models are transported in optimum conditions like on agarose-based solidified medium with growth and maintenance media to ensure appropriate viability of the reconstructed models and the high barrier functions and metabolic activity required for specific safety and efficacy testing.

### C. Transferred skin tissue on chip

One of the most straightforward approaches to generating an SoC model is to transfer the skin tissue (human or animal biopsy or human skin equivalent generated *in vitro*) to a microfluidic device (Fig. 4).

The main advantage of this approach is a more “*in vivo*-like” structure due to a better mimic of the skin structure being functional in studies such as drug diffusion, toxicity, drug sensitivity, and skin irritation. In addition to using commercially available skin models (EpiDerm™) on microfluidic structures for transdermal drug testing,^86^ other solutions were developed. An example is the work of Kim *et al.*,^80^ which proposed an SoC device for studying neutrophil responses (suspended in the whole blood) to bacterial infections in a full-thickness human skin tissue sample. The device comprised two compartments: one for the loading the skin tissue sample and one for whole blood circulation, connected through a migration channel and a filter to retain the red blood cells. The device was tested for antibiotic screening. An HSE model was proposed by Abaci *et al.*^83^ with an alginate sacrificial layer that mimics the vascularization generated using casting in a 3D-printed mold. The cell structure was built above the sacrificial layer (dermal fibroblast suspended in a collagen followed by seeding keratinocytes). The cell structure undergoes epidermalization and cornification before dissolving the sacrificial layer. The structure was used in a perfused microfluidic chip. The exciting approach uses the sacrificial layer to mimic the vascularization with potential application on topical and systemic transdermal drug delivery.

### D. Skin models

#### 1. 3D bioprinting of skin replicates

3D printing-like techniques were used to join cells, growth factors, bio-inks, and biomaterials to fabricate biologically relevant human parts replicating natural tissue characteristics. One of the critical elements of the 3D bioprinting process is the bioink. There are several requirements regarding the bioink properties. The first essential element is to assure the printability of the cells, having a specific viscosity to ensure easy extrusion through the printing head. Moreover, the bioink must be biocompatible to guarantee cell viability during the printing process (reducing the shear stress) and after printing (for cell proliferation). For this last reason, the bioink must incorporate grow factors, generating a microenvironment for promoting skin tissue regeneration. Other important aspects are related to mechanical stability and good biodegradability.^87^ Different materials were used for 3D bioprinting of skin: natural polymers such as gelatin,^88^ collagen,^89^ chitostan,^90^ alginate,^91^ fibrin^92^ or syntenic polymers like PEG,^93^ PLA,^94^ and PCL.^95^ Natural polymers present good biocompatibility and biodegradability but low immunogenicity and poor mechanical properties. Conversely, synthetic polymers present controllable mechanical properties and stability. Combinations of natural and synthetic polymers are often used to better control materials′ properties.^96^ As for the 3D bioprinting method, another critical aspect of the common technique is extrusion.^88^ Inkjet printing (droplet based printing^94^ or continuous printing^95^) was also used.

In an initial work, Lee *et al.*^97^ proposed a layer-layer skin structure fabricated by 3D printing. The proposed skin model consists of alternating printing of six collagen layers and three fibroblast layers followed by two keratinocyte layers embedded in collagen. The culture cycle was 3 weeks, the first 8 days in a submerged culture and 2 weeks under air-liquid interface ALI) conditions. A drastic reduction of the thickness of the skin structure was observed once the air-liquid interface was generated (∼50%). Moreover, the thickness of the cell culture decreased over time. From a histological point of view, during ALI exposure, the tissue became more transparent, indicating stratum corneum formation (corneocytes are formed through differentiation from keratinocytes), and a quite dense but thin (50 *μ*m) epidermal layer. At the same time, the dermal region is less populated, probably due to the collagen layer that limited the nutrients′ access to the deep of the artificial structures. In another work, Jin *et al.*^98^ used inkjet printing, a gelatin-based bio-ink and cells isolated from mouse skin to construct a skin model using ALI microenvironment to underline role of calcium ions in HaCaT cell differentiation. Kim *et al.*^99^ developed a skin-derived ECM bio-ink for 3D-printing prevascularized skin patches aiming at *in vivo* wound healing. The *in vivo* results showed that the 3D-printed patches loaded with endothelial progenitor cells and adipose-derived stem cells induced wound healing through reepithelization and neovascularization. An overview of the technologies for 3D printing of skin grafts is presented by Balavigneswaran *et al.*,^100^ which presents the design requirements, and limitations involved in the generation of skin grafts with a particular focus on the selection of the bioink.

#### 2. Commercially available skin models

The past 10 years identified technological criteria that impact the process and acted or still act like milestones. Numerous models have been proposed and presented as viable solutions only few are FDA-approved.

**FIG. 5.**
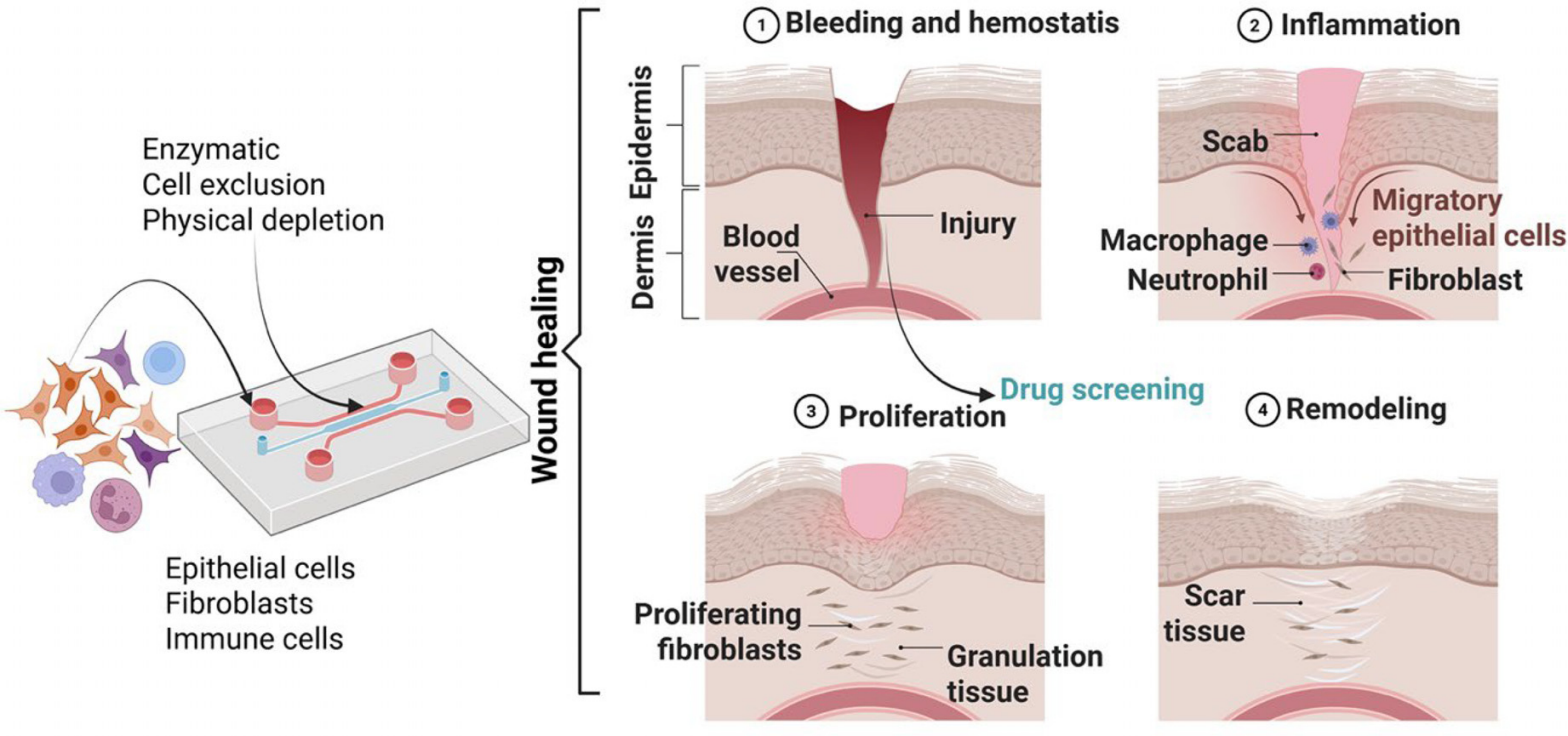
SoC model for wound healing comprising the device with the essential cells and the enzymatic processes to mimic the histological details in skin tissues repair and wound healing (skin injury with bleeding and hemostasis, inflammatory, proliferative and remodeling phases).

Their main advantage is the complex design level, which assumes the respective metabolic functions for accurate evaluations. The essential challenges stem from the desired complex functionality and standardization for consistent results. However, the challenges tissue-like assemblies faced are primarily related to the controlled replication and metabolic reconstruction of the skin barrier and subunits 3D printing as ready-to-use elements of the skin platforms. The FDA-approved models′ clinical applications target wound healing (Fig. 5). For instance, **Apligraf** (Graftskin, by Organogenesis Inc., Canton, MA, USA and Novartis Pharmaceuticals Corporation, East Hanover, New Jersey, NJ, USA) is commercially available for *in vivo* applications for clinical skin replacements and grafts. Bioengineered with living cells, it is a skin substitute, as a “composite skin graft,” “skin equivalent” or “organo-typical skin substitute,” comprising allogeneic living epidermis and dermis cells. In Apligraf, fibroblasts and collagen are combined in a dermal matrix used as a scaffold for the seeded keratinocytes generating an epidermal layer. In 1998, the system obtained FDA approval, being the first proper complex skin graft with clinical indications in treating venous or neuropathic diabetic ulcers. It is used in general, for wound dressings.^101–103^

**OrCel** is a bilayered cellular matrix and natural skin substitute (manufactured by Ortec International Inc., New York, NY, USA). It comprises a Type I bovine collagen sponge with two separate layers of cultured epidermal keratinocytes and dermal fibroblasts (normal human allogeneic skin cells). Like Apligraf, this construct represents a Dermo-Epidermal substitute for wound dressings for *in vivo* applications.^101,102,104^ Compared with these two models, **Epicel**, also called “Cultured Epidermal Autograft” or “CEA” (by Vericel Corporation, Massachusetts Institute of Technology, USA) is a graft made from the patient's skin. These grafts are indicated as a permanent skin replacement to patients suffering from deep dermal or full-thickness burns. Upon skin biopsy, the autologous keratinocytes are isolated *in vitro* and then cultured so they will form a sheet ready to be applied to the wound bed. This therapeutic approach has been successfully applied to thousands of burn patients.^102^ Epicel is the only treatment of this type (Natural skin substitute -allogenic cells) approved by the Food and Drug Administration (FDA) for use in the United States, having the methodology pioneered in 1981. Epicel, with cultured epidermal autografts, is clinically authorized and prescribed to adult and pediatric second and third degree burn patients (at least 30% total body surface area).^101,102^

Similarly, **Recell/CellSpray** (Spray-On Skin Cells, by AVITA Medical Americas, Inc, Valencia, USA) was approved for treating Full-Thickness Skin Defects (severe burns) in 2018. This system is a stand-alone, rapid, autologous cell harvesting device able to use a small skin sample and prepare, produce, and deliver an epithelial autograft suspension of single living cells and stimulate healing and repigmentation. It works through few steps. First, the skin biopsy, followed by the *in vitro* cell culture. The most actively proliferating cells are harvested, and the spray delivers suspended subconfluent autologous keratinocytes. Notably, this system also uses cultured or noncultured cells and has a reduced cell culture turn turnaround time and is indicated to treat partial thickness and graft donor sites. However, CellSpray must be combined with a dermal layer to act as a permanent skin replacement in full-thickness wound treatment.^105,106^

## IV. APPLICATIONS OF SOC

### A. Advantages and challenges of the microscale skin models

The utility of SoC in preclinical testing of drugs and cosmetics is one of the most appealing applications.^107^ Therefore, SoC-based testing should include toxicokinetic (TK) and toxicodynamic (TD) evaluation. The TK studies are necessary when the “ingredients” can reach the systemic circulation and exceed a specific plasma concentration (minimum toxic level) critical for the user's safety. These studies focus on the mechanisms that lead to toxic effects and the toxic effects of chemicals on various cells. The TD studies may help understand the active ingredients' mechanisms of action at the molecular level and determine their efficacy and potency. Despite a lack of consensus on the simulating capacity of an artificial skin model,^108^ results showed that several reconstructed skin models are reproducible and consistent in the experimental results. Notably, these models may be used to evaluate pharmaceutical/cosmetic products or in modeling pathological skin conditions, in addition to *permeability*, *irritation*, *corrosion*, *hydration*, *genotoxicity*, and *absorption.*^109^

By integrating 3D tissue, SoC platforms present adjustable microenvironments capable of mimicking *in vivo* conditions, thus compensating for the 2D and 3D models' lack of multiscale tissue–tissue or organ–organ interfaces linked to physiological cellular biomechanical indicators (stretch or shear stress). Another advantage is the micro-scaled skin models: the up to 36-fold reduced number of cells and culture media with physiologically customized fluid-to-tissue ratios.^110^ Furthermore, embedded microsensors allow the SoC record essential diagnostic parameters in real-time, allowing reconfiguring the drug testing procedures.^86,111–114^ However, mimicking the skin′s extracellular matrix and its integration with cells on the chip is challenging. One example is how collagen shrinks while fibroblasts proliferate and detach from the supporting membrane on the chip,^115,116^ also influencing skin permeability.^117^ Skin viability also depends on the SoC modeling parameters.^118^ One commercial model by Biosolution Co., Ltd., Seoul, South Korea, referenced 20 chemicals on a human epidermis model reconstructed with Asian skin tissue.^119,120^

Therefore, it is challenging for the commercial epidermis, pigmented skin, and full-thickness skin models to reach a common ground and develop reference models that meet the genetically keyed-in human skin intra- and inter-variability.

### B. Drug screening

Pharmacological testing includes pharmaco-dynamic and -kinetic testing toxicokinetic evaluation. Microfabrication for biological purpose recently supported the development of microfluidic devices with micrometer-sized chambers to house and facilitate dynamic cells culture and model or mimic the physiology of interest.^121^ Simulating tissue–tissue interfaces with porous substrates dividing the microchannels^122^ on microscale tissues as organ-on-chip permits the close-to physiological parameters transmembrane transport for pharmacokinetic^110^ and toxicology studies^123,124^ at high throughput capacity, reduced reagents volumes and costs.^125^ SoC allows the flows, forces, or chemical gradients control^126^ for the culture of skin tissues for drug and cosmetic testing. However, the “arsenal” of diseases modeled in SoC systems is still limited, and design-related difficulties are evident.

The most designed microfluidic chips generated the skin models by directly introducing the tissue inside the devices. In this case, a skin fragment from biopsy or *in vitro* generated HSE were transferred on a chip to form epidermal and dermo-epidermal models. The most popular designs comprise a dermal compartment because the mature tissues offer a complete multilayer structure. The advantage resides in their clinical potential as testing devices for molecule diffusion, multiorgan crosstalk, drug sensitivity, and toxicity. One such device intended for the transdermal transport of substances evidenced itself as one drug testing support. It comprised an HSE fragment placed in a well and cultured on top of a porous membrane that permitted nutrients′ diffusion from and to a bottom channel.^110^ Despite originating from single tissue models, SoC by skin transfer developed into a method to support coculture-based microfluidic chips. Combining biopsy-collected skin and hepatic tissues directly on a microfluidic chip allowed drug toxicity testing.^81^ One essential aspect needed is the life span of the designed chip to employ the crosstalk between tissues for drug toxicity fully. Therefore, other co-culture-based models were used as multi-organ chips. One model used skin transfer from human subcutaneous tissues biopsy, hair, and a commercial bi-layered skin equivalent (EpiDermFT™) and demonstrated better viability of the dermo-epidermal component and a longer life span of the commercial model.^30^ Another more complex model, comprised skin from a human biopsy, and also intestine, liver, skin, and kidney tissues, and demonstrated high cell viability in all tissues for 28 days.^74^ Despite the successful combinations, these models were still far from the complex physiological parameters and new alternatives were considered for drug screening.

The focus shifted to generating skin directly on the chip. For instance, static and dynamic microfluidic chips were constructed on polydimethylsiloxane (PDMS) with the dermal compartment of fibroblasts embedded into collagen gel on top of a porous membrane.^127^ To further develop the dynamic conditions for testing, other drugs were considered for the same SoC model. Sorafenib, used to treat hepatocellular carcinoma,^47^ and a *Curcuma longa* extract as an antiaging drug were administered on the SoC models.^128^ For instance, PDMS was used for a photolithography-free microfluidic chip with various channels to run concomitant tests. The device, designed for cell viability and permeation analysis, included immortalized keratinocyte monolayers and tested the allergenic effect of potassium dichromate in dermatitis. The proposed design recreated the skin using only the channels as tissue culture compartments. This aspect brought the models closer to the physiological dynamics and allowed longer viability despite their lack of real-to-life architecture.^76^ Conversely, the model by Wufuer *et al.*, despite using only cell monolayers, resembled the skin′s natural architecture and through three channels to mimicked the epidermis, dermis and a blood vessel separated by porous membranes.^75^ Moreover, it used the channels for containing the tissues, mimicking inflammation, and the dexamethasone-based treatment. These examples inspired the work on viable SoC models with a 3D structure generated directly inside a device′s channels. The desired outcome is the skin equivalents with greater resemblance to natural human skin. One way to achieve it was pumping air into the upper compartment to decrease the differentiating time to two weeks from a minimum of three weeks and still obtain the dermo-epidermal junction and superior epidermal barrier.^129^ The proposed SoC models targeted skin therapy and cosmetics. For instance, one proposed model incorporating skin and liver co-cultured tissues tested the pharmacokinetics and pharmacodynamic properties of permethrin and hyperforin in a dynamic multi-organ-on-a-chip. The model also aimed to measure the gene expression of relevant xenobiotic metabolizing enzymes in the liver spheroids for the toxicodynamic evaluation.^82^ An important evaluation parameter is the SoC permeability for various molecules. One fabricated model included a peristaltic pump and silicon tubes connected to patent vascular channels (open for blood circulation) in a skin-equivalent and addressed the issue of missing perfusion due to an inaccessible spontaneously and randomly formed dermal layer. The SoC was tested for caffeine and isosorbide dinitrate, and the model morphology allowed different permeability and the drugs to reach the vascular channel and then the bottom of the skin equivalent.^130^

Another difficulty in constructing a proper SoC stems from the biological aspects of the skin. The 3D tissues of diverse layers and cells are arranged in a precise way and present significant and complex connections between the keratinocytes and fibroblasts involved in the maturation and metabolism. Therefore, verifying the correct maturation is essential. Currently, two-photon microscopy complemented with histological images or transepithelial electrical resistance (TEER) are used to complement the classical histological and immunochemistry. Such monitoring paves the way for integrated electrochemical, optical, or even physical biosensors for direct and rapid quantifying of analytes′ levels and for real-time monitoring of the skin equivalent formation or drug administration.^72^

Literature on sensitive skin syndrome treatments concluded that an appropriate 2D or 3D model to study skin sensitivity and evaluate active molecule on sensitive skin requires sensory neurons and keratinocytes to assess skin and neuron interactions. Moreover, the ideal sensitive skin model that triggers the TRPV1 pathway would be on a compartmentalized microfluidic device, with independently cultured humans derived neurons and skin cells interconnected by microchannels to fully recapitulate the anatomical structures and be able to mimic various stimuli physical (mechanical, electrical, electromagnetic), chemical (toxins), or molecular (capsaicin, oxytocin, pro-inflammatory). Essentially, the proposed models should be standardized for reproducibility and large-scale production to address conditions such as psoriasis, atopic dermatitis, eczema, wound healing, skin irritation, herpes infection, hyperhidrosis, neuropathy, and skin sensitivity syndrome.^131^ SoC models simulating inflammation for drug-based treatment is a key holder^75^ to the next chapter that connects microbiota to skin pathology.^132,133^ Toxicology testing targeting percutaneous absorption demonstrated the potential of The Phenion^®^ Full-Thickness Skin Model utilized in the comparative studies of benzoic acid, nicotine, testosterone, and caffein quantitative transdermal delivery on pig skin in Franz-type diffusion cells.^134^ The results showed that the Phenion^®^ model mimicked the human skin well, and it may be helpful in percutaneous absorption studies upon validation.

The existing commercialized models for skin conditions like psoriasis or dermatitis^34,120,135,136^ are the avenue for even more versatile future dedicated work. This work will address the aspects contributing to testing efficacy, from design to legal aspects of marketing and commercialization.

### C. Cosmetics testing

The global cosmetics market was estimated at USD 374 billion in 2023, projected to grow from USD 393 billion in 2024 to 758 billion in 2032.^137^ New cosmetics formulated attentively for better efficacy are produced continuously, and it is generally recognized that cosmetic products present low health hazard levels.^138^ Cosmetics are organized based on their targeted areas (skin, hair, nails) and purpose (skin enhancing, makeup, color, fragrances, personal), while their availability as over-the-counter (OTC) depends on the kinetics and toxicological testing.^12^ Even though standard definitions and regulatory policies are not yet in place worldwide, cosmetics are used by all age groups and by sensitive groups such as pregnant women, children, and elderly with various comorbidities, under the general agreement that the products entering the market should be safe for consumer use under normal or foreseeable conditions of use. Since it can be argued that risks may arise unless strong and consistent quality assurance and quality control accompany them, combining the toxicological, pharmacodynamics and pharmacokinetics testing methods,^139^ provides comprehensive information, profiles the cosmetic ingredients for upgraded personal care awareness worldwide and builds new approaches to safety evaluation according to the seventh Amendment of the European Cosmetics Directive 1223/2009,^140^ Registration, Evaluation, Authorization and Restriction of Chemicals (REACH),^141^ the revised Annex VII to Reg (EU) 2017/706,^142^ and the Scientific Committee on Consumer Safety (SCCS) of the EU.^143^

Consequently, traditional animal models, such as the Guinea Pig based assays (GPMT or the Buehler test)^144^ and the murine Local Lymph Node Assay (LLNA),^145^ were excluded from testing for cosmetic substances. There is a need to test skin models specific to the previously mentioned categories of commercial products^146^ to ensure the compounds' or chemicals' efficacy, permissibility, and safety levels. For instance, establishing the balance between hygiene and protection levels is crucial when testing and determining the exact effect of chemicals loaded with carboxyl head groups, which contribute to higher protein binding on the skin surface. Therefore, matching is required surfactants with various levels of skin sebum, oil-soluble skin oils or lipophilic substances of the intercellular lipids are required for personalized cleansers.^147^ Notably, specific corrosion tests used excised rat^148^ skin, while some were not adopted in European legislation^149^ or not allowed for cosmetics and their ingredients yet.^150^

Opportunities presented for building skin models for cosmetics testing, from the classical Langmuir–Blodgett films, black/bilayer lipid membranes (BLMs) and supported lipid bilayers (SLBs) to the single- or multicompartment architectures known as artificial cells.^151^ These models, while able to closely replicate normal human skin as single (epidermis) and double layers (epidermis and dermis) models, present disadvantages mainly related to their capability of distinguishing the different layers for the chemicals penetration tests^152^ or unwanted inter- and intra-batch variability.^153^ The concept of a bottom-up skin model for screening dermal absorption of cosmetics needs to be supported with careful membrane engineering via controlled lipid and protein composition, encapsulated chemical components for metabolism, and 3D tissue-like assemblies.^154^

There are validated commercially available reconstructed human epidermis models like EpiSkin™ (L'Oreal, France), EpiDerm™ (MatTek Corporation, Massachusetts, USA), SkinEthic™ (SkinEthics, France) and epiCS^®^ (CellSystems, Germany) EPiSkin™, EpiDerm™ SIT (EPI 200), SkinEthic RHE™, LabCyte EPI-Model24 SIT, epiCS^®^, Skin+^®^ f and KeraSkin™ SIT^155–157^ used to evaluate skin-cosmetics interactions such as skin corrosion, irritation, phototoxicity, genotoxicity, sensitization, and permeation.^158^ Some tests have been recognized for their merits as alternatives to the Draize test for skin irritation and corrosion and have been validated accordingly: EpiSkin^®^, as the only method to accurately differentiate skin irritants from non-irritants, SkinEthic^®^ as the only replacement for Draize rabbit *in vivo* irritancy tests^159^ and a mean to differentiate corrosives and non-corrosives.^160^ Another notable aspect of the skin irritation tests EPiTRI model,^161^ which met the OECD Criteria, is related to the existing paracrine communication between the incorporated cells (keratinocytes, fibroblasts and stem cells) within the extracellular matrix. EpiSkin^®^, used for toxicogenomic analysis as part of SENS-IS method, is under evaluation. This test addresses the complex pathway of skin sensitization and involves 62 biomarkers as sets of genes detected by RT-qPCR. It is applied topically and adapted to cosmetics and topical formulations to evaluate the activation of critical elements in the immunological pathway, such as chemical sensitizers, metabolites, and proinflammatory factors.^162^ However, for sophisticated toxicological evaluations such as irritation and hepatotoxicity, the models must compensate for the shortcomings of less sophisticated 2D and 3D skin models like cell sheets and artificial tissue-engineered skin,^163^ with fine control of the local microenvironment and significant mechanical stimulation. Both requirements are achievable with 3D bioprinting capable of providing microfluidic SoC vascularized and pigmented skin equivalents^164,165^ and immune response.^139^ Three models of 3D hybrid human skin-on-a-chip models for attentive toxicological evaluations of cosmetic and drug formulations recapitulated skin barrier sensitization or cellular toxicity: (1) the skin cornification with keratinocytes cultured at the air–liquid interface in the top layer of a vertical microfluidic chip, (2) the skin–nerve for skin sensitization, with differentiated neural stem cells in 3D collagen adjacent to and under the keratinocyte layer; and (3) the skin–liver, for hepatic toxicity with pluripotent stem cells-derived hepatocytes to quantify the glutathione and reactive oxygen species.^166^ Despite the limitations stemming from skin structural- and tolerance-to-chemicals-variability, it is one step toward standardization and regulatory requirements. For instance, HUMIMIC Chip2 (“Chip2), the first multi-organ chip technology to incorporate skin models, is under study. It permitted the topical route′ analysis, and its application is evaluated. Furthermore, the pharmacokinetic and pharmacodynamic profiles of hyperforin and permethrin parent and metabolites evaluated the metabolic capacities of EpiDerm™ and liver spheroids and the EpiDerm™ barrier function for relevant risk assessment of topically applied cosmetic formulations.^82^ Since the safety assessment includes local sensitivity to various chemicals, learning the skin and neuron interactions for a systematic evaluation and efficacious therapy of sensitive skin syndrome treatments is required. Therefore, selecting relevant and predictive *in vitro* models is needed, too. *In vitro,* microfluidics-based sensitive skin models such as a 3D compartmentalized innervated SoC (Netri™) successfully demonstrated the activated transient receptor potential V1 (TRPV1), which elicits capsaicin-induced skin sensitivity such as heat, pain or histamine-induced itching. The microfluidic technology and compartmentalization allowed the human sensory neurons and keratinocytes to be modeled as barriers with dendritic connections as co‐culture of human skin explants and sensory neurons from the dorsal root ganglia of rats.^167^ One model, constructed on a microfluidic chip as a sensory neuron-epidermal keratinocyte co-culture model, used the slope-based air-liquid interfacing culture and spatial compartmentalization. Compared with previous co-culture models, it presented several improvements such as a better organized basal-suprabasal stratification, barrier function, and physiologically relevant anatomical innervation. The features also demonstrated the *in situ* imaging and functional analysis in a cell-type-specific manner.^168^

Furthermore, a rapidly progressing research field on safety evaluation includes the percutaneous absorption toxicokinetic alternative approaches for controlled applications based on free-of-animal testing methodologies. For instance, exploring the Absorption, Distribution, Metabolism and Excretion (ADME) of any substance introduced in the body as cosmetics or medication targets the profiles for potential toxicity via *in vivo* dermal and skin absorption or *in vitro* skin absorption. One ECVAM-validated model is the well-known *in vitro* skin static or flow-through diffusion with Franz cell^169^ using human skin for permeation testing. *In vitro* and *ex vivo* permeation tests were conducted for molecules' delivery with SoC systems. A caffeine SoC platform for caffeine and Curcuma long leaf extract (CLLE) as anti-aging cosmetic compounds was tested. It included three functional compartments arranged in a specific order to mimic the permeation: the delivery above the skin equivalent, the skin sample and the receiver at the basal skin layer. The platform was validated based on testing caffeine (a hydrophilic drug model) penetration through rodent skin. The SoC platform evidenced similar results to the already Franz cells diffusion cells, and the differences were probably due to nonspecific interaction between the caffeine particle and the PDMS housing.^170^ The CLLE evaluation conducted on an SoC platform was promising. It employed a full-thickness skin equivalent cultured in a pumpless microfluidic platform. The experiment showed the efficacy and potency of CLLE as a skin antiaging agent (50 ug/ml).^128^

However, as the newly engineered *in vitro* models undergo validation and approval processes, their relevance is questioned from the histology and physiology point of view, as the model may interfere with and metabolize the tested chemicals.^171^ Each animal model contributes to the variability of intra- and inter-laboratory practices, and both can mislead the evaluation.^172,173^ Therefore, the already in place pharmaceutical miniaturized multiorgan-on-chip systems could become the micro-physiological systems of choice to overcome the physiology-related challenges.^174^ Since 2010, important steps have been taken, and models used primary keratinocytes from neonatal foreskin, abdominoplasties, or cells from older donors, immortalized keratinocytes or keratinocytes from induced Pluripotent Stem Cells. The epidermis features and functionality were obtained via differentiation, epithelial stratification, and cornification of the cells cultured in cell culture inserts and lifted to the air–liquid interphase.

Considering the tissue complexity,^175^ particularly the skin models as microfluidic chips followed simplified monolayered architecture on which cells are manually added as microbiopsies.^176^ One SoC model combined microfluidics and full-thickness human skin micro-biopsies to study of neutrophil migration to the skin in skin infections and relevant antibiotic treatment.^80^ Other models involved human liver and skin co-culture by transfer as dynamic long-term 3D microfluidic device,^81^ compared *in vitro* SoC with *ex vivo* tissue viability in dynamic skin and hair set up,^30^ or evaluate the long-term interconnections of co-cultured human intestine, liver, skin, and kidney cells on the skin equivalent model for more insights on local metabolism and kinetics.^74^ The toxicologic aspects of skin irritation were observed on dedicated 3D platforms. One platform monitored the irritative effects of ubiquity in cosmetics sodium lauryl sulfate and steartrimonium chloride via the interactions between epidermal keratinocytes and dermal fibroblast and the effect on local angiogenesis.^171^ The results showed its potential to replace animal testing for cosmetics and pharmaceuticals and be a disease model for irritant contact in dermatitis. Another model with cells on a chip compared a static skin barrier and liver toxicity for xenobiotic metabolites toxicity data (terbinafine and sodium dodecyl sulfate). The results showed that the co-culture chip was more sensitive in assessing the metabolism (albumin and enzyme expression) and the toxicity (apoptosis). Therefore, the proposed microphysiological system is a promising tool for more than a skin irritation evaluation.

The principles of tissue engineering in skin repair^177^ help generate skin models directly inside the microfluidic system. For instance, one miniature human skin model co-cultured epidermal, dermal, and endothelial cells separated by a transparent porous membrane for nutrient flow and cell communication. The *in vitro* SoC, with a replica of skin structure and functions, showed the epidermal permeation of TNF-α, the inflammatory dermal response and the drug model (Dex) diffusion toward the endothelial layer for the inflammation protection, thus demonstrating its potential as toxicity testing for cosmetics and medication.^75^ Several models were constructed to mimic the immune responses and screen the cosmetic ingredients designed as inflammation preventives or controllers. These models employed 3D dynamic cellular environments with co-cultured immortalized human keratinocytes (HaCaT) and Human leukaemic monocyte lymphoma cell line (U937)^59^ or dermal fibroblasts and keratinocytes and matured endothelial layers^178^ for even a full-size bioprinted human skin with immune cells included.^179^

New CAD methodologies could be an elegant solution and a significant milestone in developing a complete digital microenvironment for artificial tissues. They would support standardizing artificial tissues containing thousands of different interconnected artificial cells within an approved platform.^154^ Despite the commendable achievements, the difficulty still resides in the lack of common grounds regarding the versatility to address the mechanism of action (MOA) of each active ingredient in the targeted formulations on the same SoC.

### D. Wound healing models

Disruptions in the protective epithelial barrier, called cutaneous wounds, compromise the skin's primary role as shield against the external environment. After injury and the subsequent blood flow restriction through clotting, the damaged tissue undergoes a three-stage regeneration process consisting of inflammation, new tissue synthesis, and maturation.^180^ Disrupting any of these stages result in irregular or impaired wound healing, manifested as exacerbated cellular proliferation (i.e., keloids), inadequate wound closure (i.e., diabetic ulcers), and chronic wounds. Meanwhile, an overactive healing response contributes to developing nonfunctional fibrotic tissue and hindered vascularization.^181^ Therefore, it is imperative to investigate the cellular and molecular mechanisms controlling impaired cell migration and wound closure to enhance our comprehension of how to optimize the overall healing dynamics. It is also essential to investigate how the molecular mechanisms that intricately regulate the wound-healing process are influenced by therapeutic agents interacting with cutaneous cells. Diverse *in vitro* wound healing assays have been proposed to answer this demand and explore tissue regeneration processes in rapport with cellular mechanisms and therapeutic options. These assays imply creating various types of wounds (cell-free areas within cell layers), such as mechanical (scratching), thermal, laser, and electrical wounds.^182^ The scratch assay, the most popular *in vitro* model, involves manually removing cells from a cell layer with pipette tips. However, the traditional migration and wound healing assays relying on cell exclusion demonstrated various limitations related to difficult terminus detection, non-linear or uncontrolled gradients, low reproducibility or lack of automation-friendliness. Moreover, there is variability between the control and experimental samples introduced by the typical procedure that involves inserts′ manual removal and related damaged matrix coatings.^183^ Subsequently, improved new wound healing assays must address such variability, demonstrate application versatility, enable live cell imaging, facilitate integrated detailed quantitative analysis, and feature a simplified one-step protocol (Fig. 5).

Creating *in vitro* skin models that accurately reproduce the complete structural and functional properties of native skin holds significant potential as a substitute for *in vivo/ex vivo* assays in assessing new wound-healing formulations. Additionally, these systems can expedite the commercialization of such products. Integrating 3D printing with SoC models is an attractive strategy among various production techniques. This approach not only achieves a three-dimensional system that faithfully replicates the cell composition and architecture of native skin but also establishes a dynamic microenvironment within microfluidic systems.^184^

Several microfluidic on-chip wound healing assays have been proposed, and related experiments have been published. The microfluidic technologies in wound healing assays use specific designs and mechanical or chemical methods to artificially injure the tissues on the chip and induce cell migration into the newly produced acellular areas (Table III). Each system has a specific microchannel design to mimic skin wounds as cell-free areas produced by cell exclusion or depletion.

**TABLE III.** The strategies for on-chip wound healing assays.

Wounding strategy	Cells used	References
Enzymatic	HUVECs	186,188,189
	3T3 fibroblasts	190
	Murine epithelial cells	187
	Human melanoma	191
Cellular exclusion	3T3 fibroblasts	192
	Human melanoma	193
	Epithelial cells	194,195
	Vascular smooth muscle cells	196
Physical depletion	Human dermal fibroblasts	197
	HUVECs	198

Cell depletion occurs when cells are extracted from confluent monolayers, leaving the wounded area without the cells required for typical tissue architecture. As previously mentioned, thermal, electric, enzymatic, or mechanical methods can be employed to extract patches of cells from confluent monolayers and create wound models. The primary method for generating wounds in cellular monolayers typically relies on enzymatic removal through laminar flow modeling. In this approach, the interaction of enzymes with cells affects cellular dynamics at the boundaries. However, laminar flow patterning requires syringe pumps, compared with hydrostatic fluid handling systems with enhanced scalability suitable for drug screening. On the other hand, mechanical approaches for the cell-removal-based-on-chip wounds provide true-to-life conditions, despite the need for intricate designs such as additional layers, channels, and controllers for actuators. One aspect that supports these systems as wound healing models is the possible automation of the wounding process through pneumatic actuators, which act through pressure and exclude and deplete the cellular lines. Conversely, microfluidic cell exclusion assays employ a cell-blocking element (e.g., an actuated element, a temporary cover) before seeding the cells to prevent primary cell adhesion to their specific substrate. Removing this component and the subsequent cell attachment result in an injured tissue with wound-specific defects.^185^ Choosing the wounding approach is one challenging step in wound modeling. Additional challenges stem from the microdevices′ capability of analyzing wound healing. For instance, biological markers such as human endothelial cells (HUVECs) or mouse embryonic fibroblasts (3T3 fibroblasts) are extracted from the artificial wound bed and monitored to evaluate the entire process.

HUVECs, with their recognized nature, ease of handling, and commercial availability, are particularly interesting when investigating vascular microlesions and ruptures.^186,187^ Notable is the value of embryonic fibroblasts in early-stage and proof-of-principle studies and not for modeling dermal wound healing processes predictive for human wounds. Furthermore, for more comprehensive wound models predicting the complexities of wound healing, keratinocytes with dermal human fibroblasts and volatile cell populations, such as monocytes, emerge as optimal choices. These cells can eventually be integrated into advanced commercial full-thickness dermal and epidermal skin models for comprehensive wound healing assessments.

Within the intricate wound-healing process, hemostasis, inflammation, and cell proliferation emerge as pivotal phases. The SoC model stands out as a promising *in vitro* alternative to *in vivo* systems for investigating cell migration during wound healing and assessing the impact of therapeutic interventions. In a study by Biglari *et al.*,^199^ a microfluidic wound-on-chip model designed to replicate the inflammatory phase and offers valuable insights into the behavior of diverse cell types crucial for wound healing. In this research, the microfluidic wound-on-chip device was employed for high-throughput screening of anti-inflammatory compounds.

While models have been developed to evaluate tissue reactions during wound healing, their physiological relevance is often compromised by the absence of immune cells. This underscores the imperative for more sophisticated models that can better emulate human physiological responses, ultimately facilitating the replacement of animal models. *In vivo*, the skin's response to inflammation encompasses tissue-resident cells and a variety of immune cells recruited in response to pro-inflammatory signals released at the injury site.^200^ Hence, different cell types, including keratinocytes, fibroblasts, melanocytes, and macrophages, have been incorporated into skin cultures depending on the specific application.^201^ However, the strategies for inclusion, choice of scaffolds, cell sources, culture media, and culture durations exhibit considerable heterogeneity. For instance, Matriderm, a clinically approved 3D bovine acellular dermal substitute, comprises a non-cross-linked collagen (types I, III, and V) matrix supplemented by an elastin hydrolysate is applied to various skin defects and used as a dermal substitute for full-thickness burn wounds. Furthermore, its flexibility allows qualitative skin reconstruction and flap surgery of exposed joint capsules or tendons.^202–204^

Therefore, as a crucial requirement, the design complexity also marks the commercial potential of the proposed models for wound-healing skin substitutes. Skin substitutes containing living autologous cells of dermal, adipose, or epidermal origin are regarded as advanced therapy medicinal products (ATMPs) under EU regulations or as Human cells, tissues, and Cellular and Tissue-based Products (HCT/P) under US regulations.^205^ ATMP and HCT/P products must adhere to Good Manufacturing Practice (GMP) conditions, adding complexity to the regulatory process and increasing manufacturing costs.

**FIG. 6.**
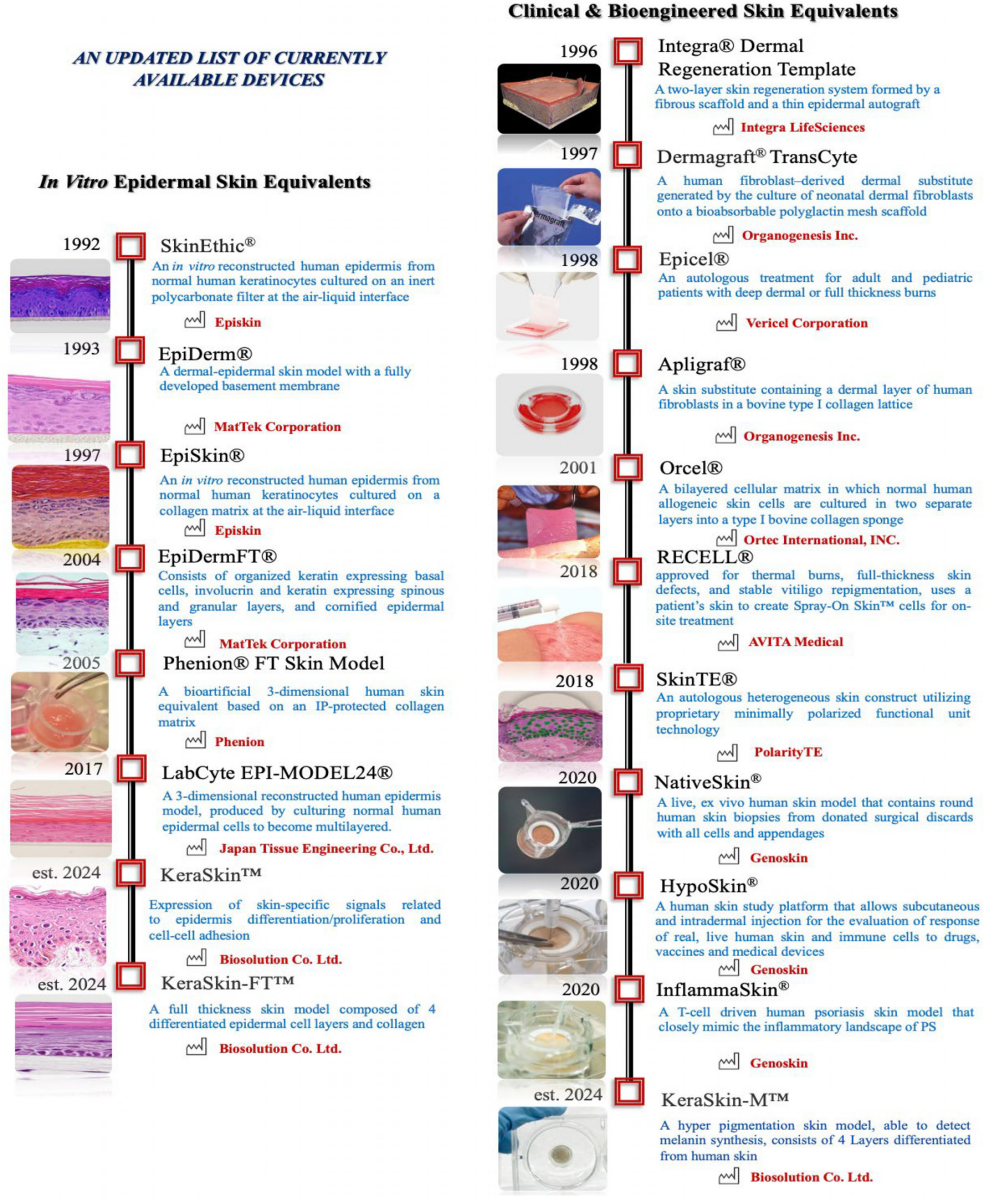
The concept of SoC and its implementation - A timeline to highlight the premarket FDA approved SoC models.

Considering the present achievements and challenges, the future designs for SoC will study assembling 3D printed artificial cells with membranal lipid compositions and synthetic metabolisms well controlled by the users. The road from the bottom-up synthetic biology principles of designing the SoC to creating state-of-the-art devices for *in vitro* dermal absorption testing is paved, despite all challenges, with technological possibilities that could lead to integrated synthetic skin models for pharmaceutical testing and clinical applications (Fig. 6).

### E. Models of chronic skin autoimmune diseases

SoC aims to address the fundamental discrepancies created by traditional models and cover autoimmune skin conditions, wound healing, infectious diseases, drug toxicity, ageing, and antiageing.^206^ The constant rise in incidence of the skin autoimmune diseases^207^ triggered the recent technological advancements in SoC devices developing and skin bioengineering to mimic the human skin environment and immune cell interactions better and allow various *in vitro* and *in vivo* models to study chronic skin autoimmune diseases such as Psoriasis, Vitiligo, Scleroderma, Lupus, Lichen Planus, Dermatitis Herpetiformis, Vasculitis, Pemphigus, Behçet's Disease, Alopecia. These models range from simple 2D cell cultures to complex 3D skin equivalents and animal models, each offering unique advantages and limitations. However, the existing 3D skin models for scleroderma, psoriasis, lupus or atopic dermatitis need to be further developed for more dynamic control.^208^ Skin-on-chip models, utilizing microfluidics technology, provide enhanced dynamic control over the environment and fluid flow, offering a more accurate simulation.^72^

*Ex vivo* research models derived from full-thickness biopsies of lesions or healthy skin are valuable tools for modeling atopic dermatitis. A photolithography-free microfluidic chip based on PDMS contained several channels for parallel experiments on immortalized keratinocyte monolayer viability and permeation assays and tested the effect of an allergen on dermatitis (potassium dichromate) on the permeability of the monolayer.^76^ Earlier on, a chip^75^ modeled inflammatory processes by introducing TFN-α through the middle channel and studied the protective effect of dexamethasone as a treatment against inflammation, giving the chip a role in drug testing.

Psoriasis explants cultured for up to 96 h retain their architecture and allow T-cell activation via the IL-23/CD3/CD28, demonstrating cytokine profiles and epidermal hyperplasia that can be modified by corticosteroids or IL-17A blockade.^209^ The InflammaSkin^®^ system activates Th17 skin-resident cells *in situ*, while maintaining IL-17, IL-22, IFN-γ, and epidermal hyperplasia in an intact tissue model for seven days.^210^ Applying imiquimod to *ex vivo* human skin causes psoriasis-like lesions and induces IL-17A expression by Langerhans cells and T cells, which can be reversed with anti-IL-17A treatments.^211^ Similarly, barrier impairment and spongiosis characteristic of atopic dermatitis were obtained after exposing explants to inflammatory stimuli, such as PMA, LL-37, or IL-4/IL-13.^212^ Gu *et al.* reported increased TSLP expression and compromised barrier function in skin equivalents for AD created with poly(I:C) and LPS, thus facilitating topical treatment testing.^213^ The inhibition of EGFR in explants led to early TSLP-mediated immune responses, therefore providing a platform for therapeutic interventions.^214^

Alopecia research involved the skin appendage-on-a-chip method, with a successful viability of the skin appendage (the hair follicle) in an optimal environment. The results published in 2013^30^ were subsequently consolidated with those published in 2023.^215^ The study utilized an extracellular matrix structured from a porous polystyrene scaffold rather than rat tail collagen type 1. However, despite the significant procedural advancement, further research is required to link the complexity of human pathology to the evolving technology for free-of-animal-models diagnostic methods and personalized medicine.

SoC has been a valuable tool for studying skin infections, aiding in their diagnosis and treatment. For instance, observing neutrophils' responses to Staphylococcus aureus on one SoC platform provided a rapid response (8 h compared with 14 h) and showed that this method is a reliable biomarker of bacterial skin infection. Moreover, using the proposed SoC for assessing broad-spectrum antibiotic treatment (e.g., penicillin) suggested its practical application for individualized antibiotic dosages in cellulitis diagnostic and monitoring.^80^ Similarly, viral infections could be modeled on SoC to study infected human skin and related treatment. An SoC model^216^ incorporated donated skin tissue and vascularized microfluidic channels to simulate the skin injuries in Human Simplex Virus (HSV) infection. The model observed real-time neutrophil migration as an infection biomarker and studied the effectiveness of antiviral drugs (e.g., acyclovir), demonstrating its diagnostic and drug screening value.

Extensively reported in 2024, skin-on-a-chip platforms are known to mimic the dynamic physiology of human skin, trying to recapitulate vascular, immune, and barrier elements relevant to infection, autoimmune responses, and disease modeling. However, validation and standardization of these types of devices are still under way,^206^ with standards such as ISO/WD 25693^217^ and ISO/WD 25448^218^ under development. Furthermore, the future directions will encompass integrating sensors into SoC for real-time monitoring^219^ and tailoring the integrated SoC platforms for personalized medicine.^118^

## V. CHALLENGES AND PERSPECTIVE

Over the past few years, research focused on improving *in vitro* skin models structurally and functionally and resulted in moving away from 2D cell models toward 3D culture systems influenced by the technological advancements. 3D bioprinting and organ-on-a-chip contribute to a better skin reconstruction and devices′ analysis power.

For instance, 3D bioprinting, through a user-generated design, facilitates layer-by-layer skin reconstruction by exact deposition of cell-laden gels designed to copy native extracellular matrices (ECMs). Furthermore, through microfluidics OoCs replicate vascularization to engineer a physiologically representative microenvironment. Also, using elastomeric substrates to create OoC devices allows transferring the biomechanical stimuli to replicate breathing,^220^ peristaltic movements,^122^ and stretching.^221^ Therefore, multiple OoC devices can be coupled as body-on-a-chip to model the systemic exposures^222^ such as toxicity screening,^223^ skin irritation,^171^ and inflammation.^75^ However, engineering an animal-free platform for dermal absorption screening is a true challenge mainly because of difficulties in replicating the barrier functions *in vitro*, in reconstituting metabolism, and the constant comparison with the previous results obtained from animal testing.

Skin models play a pivotal role in advancing dermatological research, contributing to our understanding of skin physiology, diseases, and the development of therapeutic interventions. Some skin models are used in research to test active substances and cosmetics. This group includes *ex vivo* skin models, 2D and 3D Skin Models, OoC Models, and Bioengineered Skin. A common advantage of all these is that they are more cost-effective and present fewer ethical considerations than corresponding animal models.^224^

*Ex vivo* skin models are tissue from donors or animals for *in vitro* experiments. They models are closer to the natural skin structure. *Ex vivo* skin culture can reproduce the donor particularities, including age and ethnicity, but it may lack some dynamic features of living skin.^225^

2D and 3D skin models aim to mimic the complex structure of human skin, often using layers of cells to better represent the *in vivo* environment. A 3D bioprinted skin model produces volumetric objects, adding materials layer by layer. This method has risen in popularity today because of some advantages over traditional methods, like the possibility of producing more complex structures with living cells, low costs, excellent reproducibility, and efficiency.^226^

OoC models are microfluidic devices that simulate the physiological conditions of human organs, including the skin. These models can represent skin behavior more dynamically and promise a better predictive ability in choosing an active substance that can be effective in human disease or that can fail clinical trials.^118^

Bioengineered Skin is cultured skin substitutes or grafts created using a combination of cells and biomaterials. These can be used for transplantation, as models for studying skin biology and pathologies, and for evaluating new drugs. Until today, this method cannot replicate the anatomy and physiology of normal skin.^227^

However, despite significant progress, current skin models face challenges that limit their accuracy, complexity, and translational potential. An essential challenge in developing a skin model is the complexity of human skin, with various layers, structures, functions, and variability based on age and genetic particularities. The skin exhibits heterogeneity in cell types and tissue organization. Creating models representing this heterogeneity is complex, and variations in individual skin types add to the complexity.

Proper vascularization is essential for nutrient supply, waste removal, and overall skin health. Developing models accurately representing the intricate network of blood vessels and microvasculature in the skin is a significant problem. More than that, modeling the sensory functions and innervation of the skin is complex. The integration of nerve endings and the representation of sensory responses to stimuli are crucial for understanding various skin conditions and developing effective treatments.

Skin is constantly exposed to various external factors (UV radiation, pollutants, microorganisms). Its adaptation to external stimuli depends on biomechanical proprieties. Mimicking the dynamic interaction between the skin and these external elements and integrating these factors into models to simulate realistic scenarios are complex.

Despite their benefits and research applicability, one of the greatest needs is standardization to ensure that results are comparable between different models. Nowadays, the validation process can be difficult and expensive, which has been an obstacle in their adoption and ensuring that they can replace animal testing models.^228^ Such challenges motivate the further research toward alternative materials and methods for new skin models in dermal absorption, transdermal drugs, and cosmetics delivery. There are few ways to overcome the obstructions by using artificial cells, by precisely control the lipid composition of membranes, by encapsulating chemical reagents or IVTT systems to evidence a synthetic metabolism, and by creating tissue-like assemblies from individual 3D constructed subunits.

On a positive note, the FDA put in place a comprehensive plan to evaluate new testing technology for human organ systems. Their 2022 Modernization 2.0 Act led to eliminating the mandatory inquiry of animal testing on every drug development protocol.^229^ Therefore, a solid ground that encourages advancement in *tissue-on- a-chip* technology is formed, providing reason and actual applicability for creating accurate models for human biology and disease and holding great promise for the future of personalized medicine and drug development. The future of constructing artificial tissues holds great promise. However, it depends on developing biomanufacturing strategies and infrastructure for countless applications of the synthetic skin models for dermal absorption either for pharmacodynamic, pharmacokinetic, or toxicologic screening. The future could mean the same process will generate personalized skin disease models, skin grafts, and drug or cosmetics screening platforms.

## VI. CONCLUSIONS

Although the domain of using SoC platforms to design biomimetic skin is still young, the potential is evident. These systems can replicate illnesses and bacterial infections and test therapeutic agents via toxicity and efficacy evaluations. However, developing the domain and bringing consistent and reliable results takes time and requires systematic approaches. For example, co-culturing different tissues can be considered to study the topical agents′ pharmacodynamics. Furthermore, tissue engineering and microfluidics improve constantly and must evolve to successfully translate and implement clinically promising SoC models. Solving the present technological and biological problems will improve the design, manufacturability, and usability to implement SoC devices and transform them into mainstream laboratory instruments. It is acknowledged that traditional lithography for prototyping polydimethylsiloxane (PDMS) devices reduces the industrial potential. Therefore, technological substitutes such as micropatterning techniques and materials such as poly (methyl methacrylate) or PMMA, polyvinyl chloride, PVC, or polycarbonate might be helpful. Furthermore, standardizing the chip design, materials, and manufacturing techniques should be implemented to simplify these systems′ cross-lab validation. Standardizing SoC manufacturing is critical and would integrate the platforms with existing technology and equipment for controllable fabrication, better compatibility with different laboratory equipment and precise implementation into healthcare systems. However, the financial aspects intervene and must be evaluated. Decisively, mass production is a solution. Therefore, making these devices self-sufficient, resilient, relatively simple to operate, and ready for different tissues and cells in parallel culture could reduce production costs. Also, integrating various sensors should be considered, depending on the goal and the research context.

For instance, in the pharmaceutical field, specifically drug testing, SoC devices should be integrated with sensors to monitor multiple parameters in real-time. A vital parameter to follow is trans-epithelial electric resistance, which measures skin barrier integrity and observes quality control to monitor the therapeutic or toxic effects.

The biophysical properties of the natural environment should also be observed when it comes to the biological components of the SoC platform (the dermal and epidermal compartments) during cell culture. When the dermal compartment′s properties remain constant, the risk of contraction or detachment from the culture inserts decreases. Achieving the constant properties is possible by modifying the chemical or physical properties of natural polymers for stable and physiologically relevant structures (e.g., combining fibrinogen and polyethylene glycol). An alternate path could be stimulating the dermal cells to produce their own ECM, leading to the discontinued use of animal-derived materials.^230^ Furthermore, changing the cell seeding technique from manually pipetting the cells into the device to automating and standardizing the cell/ECM matrix forming and loading process would increase efficiency.

The cell types and their origins are other parameters to be considered relevant to forming a full-thickness skin model. These parameters are the paving specifications for personalized medicine. It is acknowledged that primary cells can generate full-thickness skin models with *in vivo*-like design and that the immortalized human N/TERT keratinocytes can stratify like the primary keratinocytes. Therefore, immortalized cells should increase the reproducibility of the models. The biomimicry level of the current models could be much higher with the suitable cells and appendages integrated into the SoC. Cell types such as melanocytes, Merkel cells, immune cells, and appendages such as sweat glands should also be incorporated to increase the SoC models′ clinical potential.

Another important aspect relevant to personalized medicine is sampling induced pluripotent stem cells (iPSCs) from patients. These cells could lead to better modeling of patient-tailored disease characteristics. However, considering the high cost, low yield and reproducibility, an automation process could be integrated as a parallel process. Finally, animal serum-free perfusing media for SoC could reduce the batch variability issues associated with animal-derived materials. Since, developing physiologically significant SoC models is decisive in pharmacology and clinical research, SoC-based models will help us understand the skin-targeting therapeutic agents′ pharmacological and clinical profiles if connected with other organs-on-chips into multi-OoCs. Additionally, SoCs as models of pathological skin conditions (e.g., skin cancer) will increase the knowledge of skin biology and contribute to designing better medication for personalized medicine. Therefore, the SoC clinical and commercial translation depends on developing convergent biomanufacturing strategies and infrastructure focused on applications such as personalized skin disease models, skin grafts, and drug or cosmetics screening platforms.

## Data Availability

Data sharing is not applicable to this article as no new data were created or analyzed in this study.
